# IVF-induced pregnancy and early motherhood among women with a history of severe eating disorders

**DOI:** 10.3389/fpsyg.2023.1126941

**Published:** 2023-04-17

**Authors:** Bente Sommerfeldt, Finn Skårderud, Ingela Lundin Kvalem, Kjersti S. Gulliksen, Arne Holte

**Affiliations:** ^1^Institute of Eating Disorders, Oslo, Norway; ^2^Department of Psychology, Faculty of Social Sciences, University of Oslo, Oslo, Norway; ^3^Faculty of Health Sciences, University of Southern Denmark, Odense, Denmark; ^4^Faculty of Health and Sport Sciences, University of Agder, Kristiansand, Norway; ^5^The Norwegian Psychological Association, Oslo, Norway; ^6^Norwegian Institute of Public Health (NIPH), Oslo, Norway

**Keywords:** anorexia nervosa, infertility, anxiety, sexuality, shame, pregnancy, postpartum, IVF

## Abstract

**Background:**

There is a higher prevalence of eating disorders among women seeking *in vitro* fertilization (IVF). Women with a history of eating disorders may be particularly vulnerable to eating disorder relapse during IVF, pregnancy, and early motherhood. The experience of these women during this process has hardly been studied scientifically, despite its high clinical relevance. The overall aim of this study is to describe how women with a history of eating disorders experience the process of becoming a mother through IVF, pregnancy, and the postpartum period.

**Methods:**

We recruited women with a history of severe anorexia nervosa who had undergone IVF (*n* = 7) at public family health centers in Norway. Semi-openly, the participants were interviewed extensively first during pregnancy, and then 6 months after birth. The 14 narratives were analyzed using interpretative phenomenological analyses (IPA). All participants were required to complete the Eating Disorder Examination Questionnaire (EDE-Q) and were diagnosed (DSM-5) by using the Eating Disorder Examination (EDE), during both pregnancy and postpartum.

**Results:**

All participants experienced a relapse of an eating disorder during IVF. They perceived IVF, pregnancy, and early motherhood to be overwhelming, confusing, a source of severe loss of control, and a source of body alienation. There were four core phenomena that were reported that were strikingly similar across all participants: “anxiousness and fear,” “shame and guilt,” “sexual maladjustment,” and “non-disclosure of eating problems.” These phenomena persisted continuously throughout IVF, pregnancy, and motherhood.

**Conclusion:**

Women with a history of severe eating disorders are highly susceptible to relapse when undergoing IVF, pregnancy, and early motherhood. The process of IVF is experienced as extremely demanding and provoking. There is evidence that eating problems, purging, over-exercising, anxiousness and fear, shame and guilt, sexual maladjustment, and non-disclosure of eating problems continue throughout IVF, pregnancy, and the early years of motherhood. Therefore, it is necessary for healthcare workers providing services to women undergoing IVF to be attentive and intervene when they suspect a history of eating disorders.

## 1. Introduction

This study examines how women with a history of severe eating disorders who have undergone *in vitro* fertilization (IVF), experience IVF, pregnancy, and early motherhood. Although this knowledge is of high clinical importance to various healthcare professionals, this has rarely been examined.

Infertility presents a complex set of biological, psychological, social, and ethical issues for women, including threats to their own (McMahon et al., 1999; Chachamovich et al., 2010; Massarotti et al., 2019) and their partner’s sexual and mental wellbeing (Burns and Priebe, 1996; Keramat et al., 2014).

*In vitro* fertilization (IVF) is the most effective form of assisted reproductive technology, even though there is no guarantee that an IVF procedure will result in a child (Jain and Singh, 2022). IVF regulates hormones and stimulates the process of maturation of more eggs. Assisted fertilization refers to the process in which the egg is fertilized outside of the body. This procedure can be performed using the couple’s own eggs and sperm. The mature eggs are retrieved from the ovaries and fertilized by sperm in a laboratory. Following fertilization, the fertilized egg (embryo) or eggs (embryos) are transferred to the uterus. It takes approximately 3 weeks to complete a full cycle of IVF.

Women who have achieved pregnancy through IVF may experience it as a prolonged and difficult process that has a significant influence on their pregnancy and early stages of motherhood (Hammarberg et al., 2008; Massarotti et al., 2019). This is a challenging period in a woman’s life. It has a profound impact on their quality of life, as well as the quality of life of their partner (McMahon et al., 1999).

Women who have undergone IVF seem to be more anxious about the outcome of their pregnancy (McMahon et al., 1999; Hammarberg et al., 2008) and the wellbeing of their children (McMahon et al., 1997a). Women who have undergone IVF have been found to experience the pregnancy as unreal (Holditch-Davis et al., 1994; McMahon et al., 1999), have difficulty relating to the unborn child (Garner, 1985; Sandelowski et al., 1992; Burns and Priebe, 1996; McMahon et al., 1999), deny mental changes (Sandelowski, 1987), avoid identifying themselves as mothers (McMahon et al., 1999; Hammarberg et al., 2008), as well as avoid information about childbirth and parenting (McMahon et al., 1997b,^1999^). These phenomena are risk factors that may interfere with a mother’s adjustment to motherhood later on (Burns and Priebe, 1996).

Women who undergo IVF seem to be at a greater risk of experiencing anxiety throughout pregnancy (McMahon et al., 1997b; Hjelmstedt et al., 2003; Stevenson et al., 2019). Moreover, IVF-mothers have been found to have an avoidant coping style and to keep feelings of anxiety, depression, and anger to themselves (McMahon et al., 1997b). This is assumed to protect them from future disappointments (Massarotti et al., 2019).

Disordered eating as well as lifelong eating disorders occur more frequently among women seeking fertility treatment compared to the general population (Freizinger et al., 2010; Easter et al., 2011; Bruneau et al., 2017; Paslakis and de Zwaan, 2019; Barbosa-Magalhaes et al., 2020; Hecht et al., 2022; Le Floch et al., 2022).

Anorexia nervosa (AN), bulimia nervosa (BN), and binge eating disorder (BED) may all cause amenorrhea, irregular menstruation, and hormonal imbalance (Crow et al., 2008; Micali et al., 2014; Kimmel et al., 2016; Hecht et al., 2022; Le Floch et al., 2022), resulting in an increased risk of infertility.

Women with a history of eating disorders are at high risk of worsening or relapse of their eating problems during pregnancy and motherhood (Arnhold et al., 2019; Sollid et al., 2021; Sommerfeldt et al., 2022). Additionally, the stressful experience of undergoing IVF increases the risk of relapsing into an eating disorder (Grilo et al., 2012; Bruneau et al., 2017). Furthermore, infertility and side effects of IVF may exacerbate eating pathology (Suthersan et al., 2011; Janas-Kozik et al., 2021) or promote disordered eating (Cavalcante et al., 2019; Hecht et al., 2022).

Many women are advised to lose weight prior to conception, which may promote disordered eating (Cavalcante et al., 2019). Advice such as to lose or to gain weight will adversely affect their pathological, though very diverse, cognitive projects (Rodino et al., 2016; Bruneau et al., 2017; Sommerfeldt et al., 2022) that characterize women with a history of eating disorders. The identification of eating pathology among IVF candidates is therefore of high importance and strongly recommended (Janas-Kozik et al., 2021; Le Floch et al., 2022).

However, it is unfortunate that women with eating pathology do not disclose their conditions to their healthcare providers (Freizinger et al., 2010; Rodino et al., 2016; Paslakis and de Zwaan, 2019). Consequently, this may limit providers’ ability to diagnose eating disorders in women seeking fertility treatment and in pregnant women (Bye et al., 2018), as well as their ability to provide appropriate medical and psychological assistance (Freizinger et al., 2010; Janas-Kozik et al., 2021).

In order to achieve individual descriptions as close to the woman’s experience as possible and to process information in a way that is manageable, we have used a qualitative longitudinal research design combining semi-open interviews with interpretative phenomenological analyses (IPA).

We addressed the following research question: How do women with a history of eating disorders who have undergone IVF experience the process of becoming a mother through IVF, pregnancy, and the postpartum period?

## 2. Materials and methods

### 2.1. Participants

This study is part of a larger study entitled, “Mummy bodies.” As a part of Mummy bodies, we conducted in-depth interviews with 24 women, first during pregnancy and then during postpartum. It was necessary for all of the 24 participants to have a self-reported history of receiving treatment for a diagnosed eating disorder within the past 10 years in order to be included in the study. The inclusion criterion for this sub-study was in addition to an IVF pregnancy. Among the 24 participants, seven had become pregnant through IVF and were included in this study. All seven participants were diagnosed with an eating disorder diagnosis at both interview points. Exclusion criteria were any psychotic symptoms.

### 2.2. Setting and procedure

The participants were recruited from the general population through the free, public, universal, routine pregnancy checks at five local family healthcare centers in the greater Oslo area of Norway.

Midwives and nurses at the health centers were thoroughly informed about the project, the inclusion and exclusion criteria, and were asked to invite potential participants. Social media, podcasts, and seminars were also used to encourage and support the recruitment of participants.

Potential participants were required to email the first author and were then informed further about the study by e-mail, accompanied by an invitation to participate. The invitation contained a detailed description of the research project, including its purposes and procedures. After consenting to provide their contact information to the researcher, no participant withdrew from the study.

Participants were contacted voluntarily based on having a history of eating disorders. They were not undergoing treatment for an eating disorder when they became pregnant. If they wanted to attend, they got the possibility to get treatment through the Institute of Eating Disorders. This was information they all received before attending the study and also when they had accepted to participate. Some of the participants, based on their history of eating disorders and treatment, contacted their therapists, while others felt they got enough support from their GP and others started receiving help through the Institute of Eating Disorders based on voluntary treatment.

Following approval of participation, the participant and the interviewer scheduled a time to meet while the participant was pregnant. Additionally, the interviewer emailed the participants to arrange a new meeting for the postpartum interview. We conducted semi-open qualitative research interviews at each of the two meetings in order to gain an understanding of how they, having a history of eating disorders, experienced IVF treatment, pregnancy, and motherhood, respectively. Following the qualitative interviews, all participants completed the Eating Disorder Examination Questionnaire (EDE-Q, Bohn and Fairburn, 2008). Finally, the diagnostic interview, Eating Disorder Examination (EDE), was administered both during pregnancy and after delivery to reflect the current DSM-5 diagnosis (American Psychiatric Association [APA], 2013).

The aim of the qualitative interview was to provide rich descriptions that were as precise and accurate as possible. Data were collected using the “Experience Interview” (Holte, 2000), a semi-open, participant-centered, strategic conversation format developed from communication theory (Littlejohn, 1999).

All interviews were conducted by the first author (BS), who is an experienced psychologist specializing in clinical psychology with no connection to the participants. Each qualitative interview lasted between 120 and 180 min and was audiotaped and transcribed verbatim by the first author (BS). Altogether, the introductory information, interview, and administration of EDE-Q and EDE took approximately two and a half hours, plus/minus 5 h for each participant over two meetings. All procedures were conducted in accordance with the Helsinki declaration, and the study was approved by the Norwegian Regional Committee for Medical Research Ethics.

### 2.3. Data analyses

Data analyses were conducted in accordance with the principles of IPA (Smith et al., 2009). With reference to the main research question, the text analyses involved several steps.

Initially, the first author (BS) became familiar with the dataset by conducting the interviews, listening to audio files, transcribing the interviews verbatim, checking the accuracy of transcripts, as well as reading and re-reading the transcripts. Concurrently, the second (FS) and fifth (AH) authors listened to the tape recordings and participated as co-readers of the transcripts and as discussants of possible interpretations.

The first author made detailed notes from the interview experience as well as thoughts and comments of potential significance in order to gain an overall impression and capture relevant and different experiences. Throughout the process, we reflected upon potential biases and preconceived ideas that we might have brought to the analyses and that could possibly influence our interpretations.

The second step involved analyzing the transcripts of the seven women who had undergone IVF. There were 14 transcripts from interviews conducted during pregnancy and postpartum.

Each text was examined according to the “bottom-up principle” (Seidel and Kelle, 1995; Richards, 2015) to reveal different themes. The researchers met to discuss the results of their analyses. During the discussion of the interviews, the team became aware of some tendencies associated with pregnancy and postpartum.

We compiled a summary of each woman’s experiences during the fertility treatment, pregnancy, and postpartum period 6 months after delivery. We then introduced a sequence structure (Smith, 1999). The emerging themes were ordered according to three phases: “IVF,” “Pregnancy,” and “Motherhood.” This structure allowed us to specifically describe the experiences associated with fertility treatment retrospectively, as well as pregnancy and postpartum in real-time. Each summary was used to identify key issues of the participants’ experiences and perspectives during their fertility treatment, pregnancy, and the postpartum period. The summaries were written in accordance with the descriptions provided by the women. We then selected quotes to ensure transparency and illustrate the experiences in the participants’ own words.

Third, we recognized that the summaries from the pregnancy and the postpartum period enriched, illuminated, and overlapped with one another. The IPA identified four common core categories of phenomena across all narratives through all three phases.

Finally, to check credibility, the analyses were regularly discussed within the research team to ensure that the themes were well represented in the data and vice versa. As a result, themes and interpretations were continuously challenged, discussed, and reassessed.

## 3. Results

We begin by reporting the characteristics of the participants. Then, we present summaries of each woman’s narrative from the three phases of becoming a mother, namely IVF, pregnancy, and early motherhood. Each experience is contextualized by the narrative according to the phase in which it occurred. Next, we present the common core phenomena across all narratives, illustrated by quotes.

We have removed any information that may reveal the identity of the women. The names of all the individuals are pseudonyms. Some experiences are cited directly from the interviews, while others are references to the statements without using direct quotes. The quotes have been selected according to the extent to which they illustrate the actual experience.

### 3.1. Participants

The mean age of the participants (*N* = 7) was 35 years (range 26−42). All the participants reported a history of AN. Moreover, one participant also reported having BN in the past.

Severity was determined not by weight (because of pregnancy), but it was conceptualized and defined by three components of severity: Persistent symptoms, long duration, and treatment history (more than 7 years). All the participants reported that they had been treated for AN within the past 10 years. All information about the participants’ history of eating disorders is based solely on their self-report. None of them were receiving specialized treatment for their eating disorders when they became pregnant. All the participants re-started specialized treatment for eating disorders during pregnancy. The duration of treatment for AN before becoming pregnant ranged from 7 to 12 years (mean of 8.6 years). Furthermore, the gestation period at the time of the interview when they became pregnant ranged from week 9 to 39. The postpartum interviews were conducted between 4 and 6 months following the delivery.

Among the women, five were first-time mothers and two were second-time mothers. After the first IVF cycle, two became pregnant, two after the third cycle, and three after the fourth cycle. Of the seven women, four had experienced abortions as a result of IVF.

During pregnancy and postpartum, all participants qualified for an EDE-assessed DSM 5-diagnosis of eating disorder. Moreover, OSFED replaces the former Eating Disorder Not Otherwise Specified (EDNOS) category in DSM-IV (American Psychiatric Association [APA], 1994). None of the participants reported having binge eating disorder (BED), and none received this diagnosis when assessed during our study. Therefore, BED is not included in this study.

The symptom pressure measured by the EDE-Q Global Score was generally high during pregnancy (Mean 4.5) and the first 6 months after birth (Mean 4.4), particularly symptoms related to body, food, and weight.

### 3.2. Case summaries

#### 3.2.1. EVA

##### 3.2.1.1. IVF

The entire time, Eva doubted whether her body was prepared to become pregnant after all those years of suffering from an eating disorder. “All the time we spent wondering whether it would work was awful. It was too much for me to handle, and my eating disorder re-emerged.”

Eva experienced rapid changes due to the hormone treatment, gained a lot of weight before the pregnancy, and experienced feelings of depersonalization. “I got really fat early on. I didn’t want to tell anyone that it was IVF in case it failed. I thought I could stay pregnant for 2 months without anyone seeing it and without feeling big. But I got a stomach even before I was pregnant.” “I had a lot of water in my body. During the hormone treatment, I felt like someone else.”

According to Eva, her intention to become pregnant was important for her attitude toward her own body. “We really wanted to have children. It has been a long journey for me and us, as a couple, considering that we have been through IVF. I had to make the effort to put on weight to increase my chances of getting pregnant. I felt that it became extra important.”

Eva felt a strong sense of guilt due to the difficulties she faced in becoming pregnant. “I’ve ruined myself all these years with my eating disorder. It’s my fault, I often think to myself.”

She believed that IVF treatment strengthened their bond as a couple. “Through IVF, it became something that we were going to achieve together. We were going to have children. No matter the cost.” The idea of starting a family and working toward a common goal made her feel safe. She felt that they became important supporters of each other. However, there was less sex outside of when they “had to.”

##### 3.2.1.2. Pregnancy

Eva felt guilty for not experiencing joy regarding her pregnancy. “I was very ambivalent about the pregnancy. I was supposed be grateful, in a way, for having become pregnant, and I felt guilty toward the little child. But I couldn’t feel anything, but that being pregnant just sucks.” She felt angry and that she had sacrificed a great deal. “If it wasn’t for him being in my belly, I wouldn’t have been so fat. I can get so angry. And then I hate my body.”

Her contempt toward her own body grew as the pregnancy progressed. “It’s not the stomach that’s the problem, but everything around. Just thinking about my body gives a feeling of disgust. Being in this body is absolutely unbearable.” To endure it, Eva often had to prepare for the time following her delivery in order to be able to recover her body to its former state prior to the hormone treatment and pregnancy. “I’ll often think that I’m going to make up for it once he’s out so that I don’t harm him. But then I also know that I will harm him if I do make up for it once he is out, as well. But I can’t look like this.”

She said that she felt connected when her partner touched her once her belly was firmer. However, it was difficult to be touched in any other place other than the belly throughout the whole pregnancy: “I get a bit nervous when he touches my thighs, I get very restless and scared. I don’t like it. I am reminded of this disgusting body. When someone touches me, I can kind of feel what they feel, in a way. I can only feel the fat.”

##### 3.2.1.3. Motherhood

Eva feared that the child will develop a problematic relationship with food and their body, as she did. As a result of growing up with a mother who struggled, she has become more conscious of food and her body. “I do not want to go through all this, including gaining weight before the pregnancy, the hormone treatment, and the pregnancy, only to have my child to become ill because of me, or to be in pain. He shouldn’t suffer because of a mother who cannot take care of herself. That’s very important to me.”

Eva had difficulty experiencing maternal feelings. Additionally, she was still consumed by thoughts about food and her body. “I don’t feel like I’m a mother. I feel like I’m too immature. I don’t quite fit this role. After all, I’m immature because I still think about my body, and would rather it just disappear. I am so childish!” “I just feel so gross. I feel like I’ve gotten these long my boobs were not like this. Yes, I think this whole closeness and touch thing is disgusting. I had to stop breastfeeding. It was too much for me.”

#### 3.2.2. Cecilie

##### 3.2.2.1. IVF

Cecilie often wondered why she couldn’t get pregnant. “The fact that we started IVF was proof that it didn’t help to have gained weight. It was a bit, yeah, disappointing in a way. That I had like sacrificed it, and still had to go through IVF.”

The physical reactions associated with IVF brought back old symptoms. “I have started doing a lot of body checking again. The hormone thing completely messed it up. I was bloated in places I wasn’t used to. Everything felt different. Very confusing.”

Cecilie described the intense guilt she feels as a result of previous abortions. “I’m scared that the miscarriages were due to me exercising too much.” She was afraid to ask anyone.

Cecilie said that, despite having struggled with long-term anorexia, no healthcare professionals asked her about her relationship with food and body during the IVF process. “The fact that no one ever asked me made me think that it wasn’t a big deal.”

Cecilie recounted that during the four attempts at fertility treatment, she experienced a decline in their sexual activity. Sex became yet another area where Cecilie felt that she was not good enough. Consequently, it became yet another arena where she needed to perform.

##### 3.2.2.2. Pregnancy

Cecilie experienced a serious relapse in symptoms during the course of IVF and pregnancy. She experienced an overwhelming fear. “I’m constantly scared that something will go wrong. I think it’s due to all the abortions I’ve had. I am afraid that he will die in the womb, and afraid that giving birth will not go well.”

In the course of her pregnancy, Cecilie had difficulty imagining and bonding with the child. “I’m terrified of loss. And I’m probably protecting myself a bit by not getting too attached to the child.”

She appeared to be unsure of herself, and recounted a great deal of guilt and shame regarding her ways of thinking and being. “It’s mostly shame about myself. I kind of thought that I had come a little further than this, and that I was healthier. I should have come a lot further. I am an adult and should have been done with these thoughts.” “Since we have wanted a child so badly, I feel like an idiot for finding it so difficult to be pregnant. It has doubled up in a way. I should be happy and really proud to be pregnant. It should make me happy looking at my pregnant stomach. Instead, I would rather hide it.”

Cecilie reported that sex and intimacy with her partner were also more challenging during pregnancy. “We have grown further apart in that area. Having problems with conceiving have made it all so mechanical. IVF has destroyed that part.” “I have also been scared that sex will harm the child. My partner has accepted that explanation. He doesn’t expect us to have sex like before.”

##### 3.2.2.3. Motherhood

Cecilie’s guilty conscience over having thoughts and feelings linked to her eating disorder continued after the birth of her child. “I’ve lost all energy and can’t think about anything other than I need to exercise and what I should eat. I am completely disconnected from my family. I feel lonely. It’s like I live on an alien planet. And that is very sad.”

Cecilie had difficulty nourishing herself adequately. “I’ve been wondering if it was such a good idea to only breastfeed. Breastfeeding is a big strain on my body. I may not be getting enough food considering that I breastfeed. I also don’t sleep much.”

Cecilie talked about the lack of reactions she has received from those around her. She lost a great deal of weight shortly after giving birth. “No one has commented on the weight loss, neither my partner nor the healthcare professionals. I have found that so confusing. I’m beginning to doubt whether the number on the scale is correct. I wonder if I’m not actually that ill. I’m terrified of gaining weight again now that I’ve managed to lose the kilos I gained during pregnancy.”

Cecilie was relieved but also worried that they are still not having sex. Her partner no longer asks about it, nor does he take the initiative. “He doesn’t touch me anymore. I often think it’s because I’m no longer attractive.”

#### 3.2.3. Annette

##### 3.2.3.1. IVF

Annette underwent three attempts of IVF before becoming pregnant. She had not started menstruating again since completing treatment for an eating disorder 2 years ago. During the IVF treatment, Annette felt insecure about her own body and functionality. “I probably didn’t feel that my body was ready for children. Maybe I was simply too thin to get pregnant.”

“My body has failed and it’s my fault for messing with it so much all these years.” Annette was extremely angry with herself. “This is completely idiotic, and I get angry because I am the way I am. Because I’m sick. And now have to go through IVF because of it.”

In spite of suffering from a long-term eating disorder, Annette was never asked about her relationship with food and her body during her first IVF attempts. However, during the last attempt, questions were asked about exercise and the amount of exercise she performed. Moreover, she was asked to reduce both the intensity and quantity. She wishes she had been informed of this sooner.

Annette found herself unable to engage in sex any longer. “Sex became yet another place where I had to perform. But I wasn’t able to do it.”

##### 3.2.3.2. Pregnancy

As a pregnant woman, Annette expressed shame over her continuing struggle with food and her body. “I’m walking around with a constant conviction that I’m going to destroy my child.” However, the frustration is also directed at the child. “I notice that occasionally I get angry at the poor child inside me, which prevents me from being able to exercise and eat the way I want.”

Her body felt alien to her. “The whole process of IVF and being pregnant has destroyed me. I can’t exercise like before. I have to eat. And my body is bulky and disgusting. I don’t recognize it. I can no longer feel my hip bones, and it worries me.”

According to Annette, her body felt bloated all the time. “It’s impossible to have sex or to be touched with a bloated stomach.”

##### 3.2.3.3. Motherhood

A planned cesarean section was performed 11 days before the due date. Following the birth of her child, Annette quickly lost weight. She was proud that her weight loss had been noticed.

She said that even during the post-natal period, she wished concerns had been addressed more clearly raised regarding her rapid weight loss.

Meanwhile, she felt tremendous shame and guilt for thinking and feeling in those kinds of eating-disordered ways. “I think it would have been difficult to admit to the public health nurse if she had asked.” After the birth, Annette did not have the time or the energy to focus on getting help for herself. Now she realizes that it would have been beneficial if she had been challenged about her thoughts and feelings.

Additionally, she was afraid that this would be associated with her being a bad mother.

They started having sex a few months after she gave birth. It felt better than before the pregnancy. “It felt good to be able to have sex again without having to connect it to whether we succeed in getting pregnant or not. The performance anxiety disappeared a bit.”

#### 3.2.4. Adele

##### 3.2.4.1. IVF

Adele underwent three IVF attempts in a short period of time. There was a lot of fluid accumulation and bodily changes. This was difficult to deal with. “I was just confused. I no longer knew how big I was or how big I was going to get.”

Adele described having suffered from severe anorexia for a long period of time. During the last year of therapy, she had a clear goal of becoming pregnant. It was necessary to reduce underweight in order to implement IVF was to be implemented. At the time this requirement was put forward, she was not undergoing treatment. She felt alone and abandoned with difficult thoughts and feelings. “Once again, I felt pressured to do something I couldn’t do on my own. I felt I had to.” It was difficult for her to meet the target weight and follow the diet plan. As a result, she started throwing up in secret.

Adele felt guilty for putting herself first when she had such a strong desire to have children. Perhaps if she had gained the weight she should have gained, these attempts could have been avoided. Adele found it difficult to be honest about how difficult it was to change her diet and weight.

She recalled that she struggled to be present when they had sex. “I was just waiting for him to come. The sooner we got it over with, the better. And then I would always hope that maybe I would get pregnant this time.”

##### 3.2.4.2. Pregnancy

Adele experienced intense shame and guilt throughout her pregnancy. “I shouldn’t be acting like this. I have become pregnant, somewhat against the odds. I should toughen up, I should have been more in control with regards to food. Throwing up is not being in control. I know it’s the wrong focus and that it’s my fault.”

She described experiencing strong bodily discomfort. She avoided touching her belly and distanced herself from her body.

Adele was extremely concerned about what other people might think of her body.

She had never experienced any joy or pleasure from having sex. “It’s good to not have to have sex. It’s completely out of the question after I got pregnant.”

##### 3.2.4.3. Motherhood

Adele had a difficult time after giving birth. It was overwhelming to deal with an eating disorder. She distanced herself from her partner and felt alone. “I can’t talk to anyone. I don’t dare talk to anyone. I’m afraid that the eating disorder will be used against me. I have no one to talk to. I don’t dare to ask for help, I don’t dare do anything anymore. I am terrified that someone will tell me that I am unfit to be a mother. Then I’ll lose everything.”

It was difficult to be there for the child. She only felt connected to the child when she was breastfeeding. In that moment, she no longer thought about food or her body. It became her sanctuary.

Sex continued to be difficult. “Now I don’t have an excuse for not looking good anymore. I can’t have sex and show myself naked to my husband. I always try to avoid going to bed at the same time as him. I always turn off the light when I’m going to undress. He’s not allowed to touch me.”

#### 3.2.5. Hailey

##### 3.2.5.1. IVF

Hailey described her experience with IVF as dramatic. “I was completely ruined by those hormones. I couldn’t handle anything.” “I started reacting negatively to everything related to do with food. When we should eat, what we should eat, how much I should eat. Everything got out of control. I became a short-tempered person who became difficult to be around.” Hailey lost control over her eating habits. She started overeating and throwing up again. It is something she was ashamed of.

Moreover, Hailey was ashamed that the eating disorder may have contributed to her inability to conceive.

“My husband has probably wondered about it too. But no one has dared to raise the topic with me.”

During the fertility treatment, sex became mechanical. It was even more difficult with IVF due to close contact and touch. “I had sex only because it was required of me, but I felt no pleasure.”

##### 3.2.5.2. Pregnancy

Hailey experienced a strong fear throughout her pregnancy: “Will it work this time?”

She said she was unable to connect with the child in her womb. “Imagine if I lose it.”

Additionally, she was afraid of not being a good enough mother.

Hailey explained the shame associated with her own behavior. “I’m an adult, and I’m ashamed that I’m still struggling with this.”

And she felt alone with her difficult feelings. “Many people probably think I’m doing just fine.” The eating disorder was her “little secret bubble.” “I don’t talk about it at all. Everyone thinks I’ve recovered, that I just love exercising. Now I’ve also become pregnant, so now people think I’m not struggling anymore.”

Hailey was unable to have sex during the pregnancy. “I try not to relate so much to my body. I turn off below here,” she said, pointing to her neck. “It’s just not possible to have sex. My body is closed for renovation.”

##### 3.2.5.3. Motherhood

The anxiety continued after giving birth. She was concerned about her son. Hailey breastfed frequently, from every half hour to every hour. She was terrified that her son will be hungry. Her concern for him was evident. She associated signs of restlessness and discomfort with hunger. As soon as he touched her chest, he calmed down. “It has become very difficult to get him to fall asleep without breastfeeding.” She was worried that her son had not gained enough weight. According to his check-ups at the health center, he was below the curve. Furthermore, she had read that being underweight as a child could lead to obesity as an adult. This worried her. Moreover, she was concerned that her eating habits may have something to do with it. She was not eating well and was afraid that her milk may not be good enough for the child.

Hailey felt a strong sense of guilt when she eats wrong or does something wrong. Her sense of failure had been exacerbated by the fact that the eating disorder had completely taken over again. “Everyone actually admired me before for what I have achieved at work and with training. But then comes the eating disorder, which is a secret thing, and with that I’m not very strong. I can no longer cope with eating. It is embarrassing.”

Hailey found it increasingly difficult to be close to her partner. She withdrew a lot and experienced sex as an obligation. “I feel that I have to have sex for him to stand to be with me at all.”

#### 3.2.6. Louise

##### 3.2.6.1. IVF

Louise had a strong desire to become pregnant. She found the IVF process to be much more challenging than she had anticipated. “In the past, the eating disorder was a lot about control and mastery. I have set myself goals and managed to reach them. But then I wasn’t able to get pregnant. I had no control. I thought I couldn’t do it. I was terrified.”

Louise felt like a failure and that she was not good enough. “I was told that my only task was to eat and rest for a few weeks in the hope that this would lead to success. But those are the two things I can’t do. It felt like I wasn’t a worthy woman. Not being able to get pregnant was my great fear during all the years I was ill.”

“Sex became only something that had to be done.”

##### 3.2.6.2. Pregnancy

Even while she was pregnant, Louise was afraid that things would go wrong.

She described her guilt over not being happy about the pregnancy. “I’m just having a hard time, actually. You’re meant to have a really nice time during the pregnancy. And I’ve fought for this pregnancy, after all. But then it’s just bleak.”

She felt bad about not being able to connect with the child in her womb. She became afraid of being a bad mother. “So now I go around worrying about a weight that is really just a trivial thing in the midst of everything, instead of focusing on the human being in my belly that is going to grow. I can’t break free from numbers, weight, and control. I can’t focus on her, but only focus on myself. What kind of a mother will I be?”

While growing up, Louise often felt pressured to eat. And she felt this pattern repeated itself during her pregnancy. She stated that she now “must eat” for the sake of the fetus and this made her uncomfortable. She felt a strong sense of shame regarding eating. Additionally, she displayed a strong fear of what the interviewer would think if she disclosed what she eats. She described it as a thick mass of butter surrounding her entire body. As a result, she felt a strong need to punish herself. In order to accomplish this, she may have chosen to avoid eating or engage in intense exercise.

Louise found that IVF and the pregnancy made her relationship with her partner, in terms of closeness and sex, more difficult. She found it difficult to relate to her own body and the fact that it changed.

##### 3.2.6.3. Motherhood

The labor was difficult, and the child had a very low weight at the time of the delivery.

Louise felt a strong sense of guilt and shame for not eating adequately during her pregnancy. The role of mother served as an external motivation for her to keep the eating disorder at bay. “If I let the eating disorder destroy my relationship with the child, I will never forgive myself.”

Furthermore, she felt a fierce contempt for her own body. The physical closeness became increasingly difficult during breastfeeding. “It was a claustrophobic feeling. At times, it felt like he was glued to me. I could hardly breathe. I felt I had no choice in the end but to switch to the bottle.”

Louise felt a deep sense of despair as a result of not being able to breastfeed. Her urge to punish herself by restricting her food intake and exercising increased.

Closeness and sex continued to be difficult during the post-natal period. “Now I curse my body because it looks the way it does. I am very aware that my belly has gotten bigger. I have different shapes than before.”

#### 3.2.7. Mette

##### 3.2.7.1. IVF

Mette considered IVF to be an extremely demanding process. “We have never quite figured out what makes us unable to get pregnant. Going through IVF has been absolutely horrible. It is very frustrating not to get what you want. I haven’t experienced any sense of control.”

Mette felt like a failure because her body was not functioning properly. She had four miscarriages and became pregnant on the fifth attempt of IVF. She often felt like a failure: “It gives me a feeling of being a total failure. And I couldn’t lose weight in the meantime and get rid of those extra kilos either.”

She distanced herself from her partner. In her opinion, there was something wrong with her, and she was unable to satisfy him. As a result, she felt compelled to pull away.

##### 3.2.7.2. Pregnancy

Mette also felt a loss of control during the pregnancy. She began throwing up during IVF, and this escalated during her pregnancy: “Once I’ve started, it’s impossible to stop.”

She struggled to connect with her child: “I need to be reminded that there is a human being inside my womb.”

Mette felt an absence of joy during the pregnancy. “I can feel a flash of joy when I feel a kick. But it passes quickly. Then I think about being fat and big and what I’m going to do about that after I give birth.”

Mette did not inform her midwife, her doctor, or anyone else about her vomiting. “It’s hard to admit that I don’t eat. But even harder to say I’m throwing up. I don’t talk to anyone about that. It’s too difficult. I am very ashamed. Just having said it here is difficult. But at the same time, it’s good to know that I’ve told someone. I think there are many people who hide these things. There is so much shame, after all.”

Mette was terrified of all the changes she was experiencing. Therefore, she avoided touching and being close to her partner for fear of what he might feel. “If he gives me a hug or holds me, he will just feel the fat.” The topic of sex was off limits throughout the entire pregnancy.

##### 3.2.7.3. Motherhood

The role of mother was challenging for Mette.

As a result, she felt ashamed. She found it difficult to say the child’s name. Her fear of connecting with the child prevented her from doing so. She did not believe that she was a good mother.

Mette said that the eating disorder had completely taken over her life. Additionally, she had lost her hope of recovering from the eating disorder during the post-natal period and had lost the courage to work on it.

After giving birth, Mette noticed that her focus was more on the child. She longed for someone to care for her.

Mette still covered herself up and found sex uncomfortable. “IVF has destroyed much of what was normal about sex. I think I can never have a normal relationship with either my body or sex again.”

### 3.3. Core phenomena

Several experiences described in the seven stories were strikingly similar. Based on the analytic processes according to IPA, the authors identified four core categories of phenomena: “Anxiousness and fear,” “Shame and guilt,” “Sexual maladjustment,” and “Non-disclosure.”

However, one woman reported that sexual intercourse became less problematic after childbirth as performance anxiety disappeared. Nevertheless, the same woman reported sex as being problematic during the first two phases (IVF and pregnancy). Additionally, another woman stated that the IVF process strengthened their relationship as a couple and that it gave them a common focus and goal. This section will continue to discuss the phenomena that were common to all informants.

#### 3.3.1. Anxiousness and fear

Throughout the process, the women found that the changes caused a high level of anxiety. It was related to anxiety about bodily changes, the fear of harming or losing the baby, and the fear of doing something wrong. Additionally, there was a strong sense of insecurity and low self-worth.

##### 3.3.1.1. Anxiousness and fear during IVF

Louise: “In the past, the eating disorder was a lot about control and mastery. I have set myself goals and managed to reach them. But then I wasn’t able to get pregnant. I had no control. I thought I couldn’t do it. I was terrified.”

##### 3.3.1.2. Anxiousness and fear during pregnancy

Cecilie: “I’m constantly scared that something will go wrong. I think it’s due to all the abortions I’ve had. I am afraid that he will die in the womb, and afraid that giving birth will not go well. The thought of losing the baby terrifies me. And I’m probably protecting myself a bit by not getting too attached to the child.”

##### 3.3.1.3. Anxiousness and fear during early motherhood

Adele: “I can’t talk to anyone. I don’t dare talk to anyone. I’m afraid that the eating disorder will be used against me. I have no one to talk to. I don’t dare to ask for help, I don’t dare do anything anymore. I am terrified that someone will tell me that I am unfit to be a mother. Then I’ll lose everything.”

#### 3.3.2. Shame and guilt

The women experienced extensive feelings of shame and guilt as a result of the IVF process. Their shame stemmed from not being able to get pregnant, from having destroyed their bodies, from harming the baby, and from having an eating disorder. They felt guilty about not being able to change their weight and eating disorder behaviors during IVF, pregnancy, and the early stages of motherhood.

##### 3.3.2.1. Shame and guilt during IVF

Eva: “I’ve ruined myself all these years with my eating disorder. It’s my fault, I often think to myself.”

##### 3.3.2.2. Shame and guilt during pregnancy

Cecilie: “Since we have wanted a child so badly, I feel like an idiot for finding it so difficult to be pregnant. It has doubled up in a way. I should be happy and really proud to be pregnant. It should make me happy looking at my pregnant stomach. Instead, I would rather hide it.”

Louise: “So now I go around worrying about a weight that is really just a trivial thing in the midst of everything, instead of focusing on the human being in my belly that is going to grow. I can’t break free from numbers, weight, and control. I can’t focus on her, but only focus on myself. What kind of a mother will I be?”

##### 3.3.2.3. Shame and guilt during early motherhood

Eva: “I don’t feel like I’m a mother. I feel like I’m too immature. I don’t quite fit with this role. After all, I’m immature because I’m still think about my body, and would rather it just disappear. I am so childish!”

#### 3.3.3. Sexual maladjustment

The whole process had significant consequences for the couples, particularly with regard to sex and intimacy. In a relatively uniform manner, each of the women experienced a negative impact on their sex life, intimacy, and sexual intercourse. Several factors contributed to the difficulty experienced in intimacy and sex, including infertility, failure to achieve, and hormonal imbalance.

##### 3.3.3.1. Sexual maladjustment during IVF

Cecilie: “We have grown further apart in that area. Having problems with conceiving, have made it all so mechanical. IVF has destroyed that part.”

##### 3.3.3.2. Sexual maladjustment during pregnancy

Annette: “The whole process of IVF and being pregnant has destroyed me. I can’t exercise like before. I have to eat. And my body is bulky and disgusting. I don’t recognize it. I can no longer feel my hip bones, and it worries me. It’s impossible to have sex or to be touched with a bloated stomach.”

##### 3.3.3.3. Sexual maladjustment during early motherhood

Adele: “Now I don’t have an excuse for not looking good anymore. I can’t have sex and show myself naked to my husband.”

#### 3.3.4. Non-disclosure

The majority of the women did not disclose their pathology to their healthcare providers. Several of them said they were never asked about their weight, body, or eating problems. They also acknowledge the shame which can hinder disclosure. Additionally, they did not spontaneously inform the IVF personnel about it. Others were instructed to gain weight and reluctantly tried to do so, considering the IVF treatment as a motivation to control their eating problems. They were worried that eating disorders would not be tolerated by others, and would be incompatible with receiving IVF and becoming pregnant.

##### 3.3.4.1. Non-disclosure during IVF

Cecilie: “The fact that no one ever asked me made me think that it wasn’t a big deal.”

Hailey: “The eating disorder was probably the reason why I did not get pregnant. My husband has probably wondered about it too. But no one has dared to raise the topic with me.”

##### 3.3.4.2. Non-disclosure during pregnancy

Hailey: “Many people probably think I’m doing just fine. The eating disorder is my ‘little secret bubble.’ I don’t talk about it at all.”

Mette: “It’s hard to admit that I don’t eat. But even harder to say I’m throwing up. I don’t talk to anyone about that. It’s too difficult. I am very ashamed.”

##### 3.3.4.3. Non-disclosure during motherhood

Annette: “I think it would have been difficult to admit to the public health nurse if she had asked.”

Hailey: “I can no longer cope with eating. It is embarrassing.”

## 4. Discussion

In this study, we investigated how women with a history of severe eating disorders experienced IVF, pregnancy, and motherhood. This was achieved by combining comprehensive in-depth interviews with IPA of 14 narratives from seven women with a history of severe AN, who had become pregnant through IVF. In spite of its high clinical relevance, to the best of our knowledge, this is the first study to examine these processes among this highly vulnerable group of women who were becoming mothers.

The results show that the participants experienced IVF and pregnancy as overwhelming and confusing, resulting in a severe loss of control and a sense of alienation from their bodies. Failure to become pregnant naturally made them feel like a failure as a woman. They attributed this failure to their own incapacity to control their eating disorder. There was an extensive feeling of shame and guilt as a result of the IVF process, leading them to question whether they themselves had destroyed their own body to such an extent that they were unable to conceive. However, a strong desire to have children led them to pursue IVF.

Furthermore, all seven participants experienced a relapse or worsening of their severe eating disorders while undergoing IVF, and all of them experienced high levels of symptoms. Some attributed the relapse of eating problems, purging, and over-exercising to the hormone treatment. Others stated that recognizing being infertile, uncertainties, and the loss of control resulting from not being able to become pregnant triggered the eating disorders.

Some studies have indicated that women with a history of eating disorders are particularly at risk of deteriorating mental health during pregnancy and early motherhood, including relapse or worsening of their eating problems (Arnhold et al., 2019; Sollid et al., 2021; Sommerfeldt et al., 2022). Furthermore, infertility, IVF, and its side effects seem to increase the risk of worsening or relapse of eating disorders (Suthersan et al., 2011; Grilo et al., 2012; Bruneau et al., 2017; Janas-Kozik et al., 2021), or of promoting disordered eating (Hecht et al., 2022). This study adds to this knowledge by demonstrating that women with a history of severe eating disorders are more likely to experience worsening or relapse of their eating problems during the IVF, i.e., even before they become pregnant.

As far as the mechanisms behind the relapse are concerned, this study focuses solely on the participants’ reports about how they experienced it. Therefore, further research is needed to clarify whether the relapse is primarily triggered by biological mechanisms, such as hormone treatment and its biological side effects, or by psychological mechanisms, such as uncertainties and loss of control.

This study identified four core phenomena. These were: (1) anxiousness and fear of bodily changes, of harming or losing the baby, and of doing something wrong; (2) shame over destroying one’s own body, and despite wanting to become a mother, suffering from eating problems again, and guilt over not being able to change weight and eating disorder behavior; (3) sexual maladjustment, including disgust regarding their own body, hatred of being touched, resistance to showing oneself naked, and the termination of sexual intercourse after becoming pregnant; and (4) non-disclosure, including withholding information about their eating disorders to healthcare providers for fear of being incompatible with IVF, pregnancy, and motherhood.

Most strikingly, these four core phenomena were prominent in or dominated both the pregnancy interview and the early motherhood interview and were present in all three phases of becoming a mother, namely IVF, pregnancy, and early motherhood. Additionally, the four core phenomena were prevalent among all the participants and in all 14 narratives.

Since this study only covers seven women and the recruitment method does not protect against selection bias, it is not unlikely that the four core phenomena are not representative of the overall population of women with a history of severe AN or severe eating disorders who undergo IVF. However, the fact that the four core phenomena were prominent in or dominated all the 14 narratives may indicate that they are likely to occur in most women with a history of severe AN when undergoing IVF. This needs to be confirmed in representative quantitative studies before any conclusions are drawn.

This unified and unambiguous pattern of core phenomena among women with a history of AN undergoing IVF contrasts with our previous study. In that study, we found that women with a history of severe eating disorders in general, attributed a very different psychological meaning to being pregnant (Sommerfeldt et al., 2022).

Pregnancy may be a particularly vulnerable time for women’s mental wellbeing. For example, fear of losing the baby, difficulties with intimacy and sex, and worry about not being a good enough mother are common concerns during this period (Burns and Priebe, 1996). Therefore, the question arises: how can we be certain that our results are not simply common concerns that most women have during pregnancy and early motherhood? They probably do to a certain extent. However, the intensity, consistency, and all the phenomena linked specifically to pathological eating behaviors, purging, and over-exercise differ substantially from what most women experience during pregnancy and early motherhood (Eberhard-Gran et al., 2014).

Can the results be attributed to IVF or factors operating in the selection for IVF as shown in several studies, regardless of eating problems (Garner, 1985; Sandelowski, 1987; Sandelowski et al., 1992; McMahon et al., 1997a,^1999^; Hammarberg et al., 2008; Massarotti et al., 2019; Stevenson et al., 2019)? Again, they probably do to a certain degree. However, the intensity, consistency, and content linked specifically to eating disorders indicate that these women are particularly vulnerable to various mental health challenges, including relapse or worsening of eating problems, when attempting to become mothers.

It is common for pregnant women to not disclose mental health issues to their healthcare providers during pregnancy (Mule et al., 2022). This may be caused by a need for normalization and a negative self-perception, fear of negative perceptions from others, lack of trust in midwives, differing expectations regarding appointments or assessment models, and time constraints (Mule et al., 2022). The same reasons for non-disclosure apply to women with a history of severe eating disorders (Freizinger et al., 2010; Paslakis and de Zwaan, 2019; Janas-Kozik et al., 2021).

In this study, the shame and guilt associated with infertility seemed to strengthen the non-disclosure of eating pathology. All the women were ashamed about not being able to change their weight and disordered behavior during IVF. They had difficulties opening up and they were not asked about it. Therefore, they did not disclose their problems related to food and body to the healthcare providers either during IVF or later.

Therefore, clinicians involved in IVF need to be particularly observant and take the necessary precautions if there is any reason to suspect that the patient has a history of a severe eating disorder. Providing women with the opportunity to disclose can reduce their specific barriers to disclosure. This includes providing adequate assessment time and offering greater continuity of midwifery care. This again is likely to be associated with greater disclosure and improved support to prevent worsening or relapse.

## 5. Strengths and weaknesses

This study has the advantage of recruiting a non-clinical sample of informants, which is challenging given that many people do not disclose their eating problems to healthcare providers. Second, this study includes comprehensive high-quality in-depth interviews, which were supervised by two experienced seniors who specialize in this particular type of qualitative technique. This provided spontaneous, coherent, and contextualized information, expressed in the women’s own words. Third, the longitudinal study design made it possible to prospectively follow each woman’s process from pregnancy to early motherhood in addition to retrospectively retrieving phenomena from her IVF treatment. Furthermore, it enabled the diagnostic and symptom assessments to be validated by repeated measurements. Fourth, the study’s external validity was strengthened by the use of standard diagnostics. Furthermore, this study included OSFED and UFED, which replaced EDNOS from earlier versions of DSM in DSM 5.

Our study has certain limitations. First, we did not include another group, for example, women without eating disorders who had undergone IVF. Second, we are unable to determine how representative the participants are of IVF-mothers with severe AN. Even though the recruitment setting was non-clinical, we cannot exclude the possibility that the recruitment process facilitated the recruitment of women undergoing IVF treatment with more salient eating problems than others. It is notable, however, that all seven women in this study experienced relapses or worsening of their eating problems during IVF treatment, which lasted throughout pregnancy and in the first 6 months after childbirth. Third, although all the participants in the analyses qualified for an eating disorder diagnosis during pregnancy, we do not know how many or which participants recovered from the diagnoses before becoming pregnant. Consequently, we could not differentiate between women who still had the diagnosis when they underwent IVF and then worsened their condition during IVF, and those who had recovered and relapsed after undergoing IVF and becoming pregnant. Fourth, we only recruited women with severe AN. Thus, our results may not be applicable to other groups with a history of eating disorders, such as milder cases of AN or BED. Finally, we did not include their partners. Their partners could have provided comparative information. Moreover, partners play an important role in supporting women during IVF, pregnancy, and early motherhood. Further research is needed to determine the needs of these partners and the roles they play.

## Data availability statement

The original contributions presented in this study are included in the article/supplementary material, further inquiries can be directed to the corresponding author.

## Ethics statement

The studies involving human participants were reviewed and approved by Regional Committees for Medical and Health Research Ethics (REC) 20th of May 2020, Reference 92665. The participants provided their written informed consent to participate in this study. Written informed consent was obtained from the individual(s) for the publication of any potentially identifiable images or data included in this article.

## Author contributions

BS, FS, and AH contributed to conception and design of the study. BS conducted the interviews, transcribed the interviews and analyzed the material, and wrote the first draft of the manuscript. AH and FS listened to the tape records and were co-readers of transcripts and as discussants about possible interpretations and wrote sections of the manuscript. All authors contributed to the manuscript revision, read, and approved the submitted version and the analyses were continuously challenged, discussed and reassessed.
