# Corrigendum: IVF-induced pregnancy and early motherhood among women with a history of severe eating disorders

**DOI:** 10.3389/fpsyg.2025.1601971

**Published:** 2025-04-17

**Authors:** Bente Sommerfeldt, Finn Skårderud, Ingela Lundin Kvalem, Kjersti S. Gulliksen, Arne Holte

**Affiliations:** ^1^Institute of Eating Disorders, Oslo, Norway; ^2^Department of Psychology, Faculty of Social Sciences, University of Oslo, Oslo, Norway; ^3^Faculty of Health Sciences, University of Southern Denmark, Odense, Denmark; ^4^Faculty of Health and Sport Sciences, University of Agder, Kristiansand, Norway; ^5^The Norwegian Psychological Association, Oslo, Norway; ^6^Norwegian Institute of Public Health (NIPH), Oslo, Norway

**Keywords:** anorexia nervosa, infertility, anxiety, sexuality, shame, pregnancy, postpartum, IVF

In the published article, there was an error in Table 1 as published. There are concerns of backwards identification risk. Table 1 should therefore be removed from the article.

In accordance with the removal of Table 1, corrections have been made to *3. Results, 3.1. Participants, Paragraphs 1 and 6*.

In *Paragraph 1*, this sentence previously stated: “The diagnoses, background information, and a brief history of each of the seven participants are presented in Table 1.” This sentence should be removed.

In *Paragraph 6*, this sentence previously stated: “As shown in Table 1, the symptom pressure measured by the EDE-Q Global Score was generally high during pregnancy and the first 6 months after birth, particularly symptoms related to body, food, and weight.” The corrected sentence appears below:

“The symptom pressure measured by the EDE-Q Global Score was generally high during pregnancy (Mean 4.5) and the first 6 months after birth (Mean 4.4), particularly symptoms related to body, food, and weight.”

The authors apologize for these errors and state that they do not change the scientific conclusions of the article in any way. The original article has been updated.

